# Halotolerant Endophytic Bacteria *Priestia flexa* 7BS3110 with Hg^2+^ Tolerance Isolated from *Avicennia germinans* in a Caribbean Mangrove from Colombia

**DOI:** 10.3390/microorganisms12091857

**Published:** 2024-09-07

**Authors:** Zamira E. Soto-Varela, Christian J. Orozco-Sánchez, Hernando José Bolívar-Anillo, José M. Martínez, Nuria Rodríguez, Natalia Consuegra-Padilla, Alfredo Robledo-Meza, Ricardo Amils

**Affiliations:** 1 Facultad de Ciencias Básicas y Biomédicas, Centro de Investigación en Biodiversidad y Cambio Climático—ADAPTIA, Universidad Simón Bolívar, Barranquilla 080002, Colombia; christian.orozco@unisimon.edu.co (C.J.O.-S.); hernando.bolivar@unisimon.edu.co (H.J.B.-A.); natalia.consuegra@unisimon.edu.co (N.C.-P.); alfredo.robledo@unisimon.edu.co (A.R.-M.); 2 Centro de Biología Molecular Severo Ochoa (CSIC-UAM), Universidad Autónoma de Madrid, Campus Cantoblanco, 28049 Madrid, Spain; jmm.lozano@cbm.csic.es (J.M.M.); nrodriguez@cbm.csic.es (N.R.); ramils@cbm.csic.es (R.A.); 3 Institute of Applied Microbiology, Justus-Liebig-University, 35392 Giessen, Germany; 4 Centro de Astrobiología (INTA-CSIC), Carretera, Ajalvir km 4, 28850 Torrejón de Ardoz, Spain

**Keywords:** heavy metal, endophytes, exopolysaccharides, mercury biosorption

## Abstract

The mangrove ecosystems of the Department of Atlántico (Colombian Caribbean) are seriously threatened by problems of hypersalinization and contamination, especially by heavy metals from the Magdalena River. The mangrove plants have developed various mechanisms to adapt to these stressful conditions, as well as the associated microbial populations that favor their growth. In the present work, the tolerance and detoxification capacity to heavy metals, especially to mercury, of a halotolerant endophytic bacterium isolated from the species *Avicennia germinans* located in the Balboa Swamp in the Department of Atlántico was characterized. Diverse microorganisms were isolated from superficially sterilized *A. germinans* leaves. Tolerance to NaCl was evaluated for each of the obtained isolates, and the most resistant was selected to assess its tolerance to Pb^2+^, Cu^2+^, Hg^2+^, Cr^3+^, Co^2+^, Ni^2+^, Zn^2+^, and Cd^2+^, many of which have been detected in high concentrations in the area of study. According to the ANI and AAI percentages, the most halotolerant strain was identified as *Priestia flexa*, named *P. flexa* 7BS3110, which was able to tolerate up to 12.5% (*w*/*v*) NaCl and presented a minimum inhibitory concentrations (MICs) of 0.25 mM for Hg, 10 mM for Pb, and 15 mM for Cr^3+^. The annotation of the *P. flexa* 7BS3110 genome revealed the presence of protein sequences associated with exopolysaccharide (EPS) production, thiol biosynthesis, specific proteins for chrome efflux, non-specific proteins for lead efflux, and processes associated with sulfur and iron homeostasis. Scanning electron microscopy (SEM) analysis showed morphological cellular changes and the transmission electron microscopy (TEM) showed an electrodense extracellular layer when exposed to 0.25 mM Hg^2+^. Due to the high tolerance of *P. flexa* 7BS3110 to Hg^2+^ and NaCl, its ability to grow when exposed to both stressors was tested, and it was able to thrive in the presence of 5% (*w*/*v*) NaCl and 0.25 mM of Hg^2+^. In addition, it was able to remove 98% of Hg^2+^ from the medium when exposed to a concentration of 14 mg/L of this metalloid. *P. flexa* 7BS3110 has the potential to bioremediate Hg^2+^ halophilic contaminated ecosystems.

## 1. Introduction

Mangroves are ecosystems located in a critical transition zone between terrestrial and marine environments. They are recognized as one of the most productive ecosystems on Earth, playing a fundamental role in climate change adaptation and mitigation [1]. These forests are composed of plant species (halophilic trees and shrubs) with morphological, physiological, and reproductive adaptations that allow them to survive at the interface between terrestrial, estuarine, and marine ecosystems in tropical and subtropical regions throughout the world [2,3]. However, these ecosystems are under pressure worldwide mainly due to anthropogenic activities [4]. Colombia has an area of mangrove forests of approximately 300,000 ha, of which more than one fourth are found on the Caribbean coast, where five species, *Avicennia germinans*, *Rhizophora mangle*, *Laguncularia racemosa*, *Conocarpus erectus*, and *Pelliciera rhizophorae*, have been reported [5]. It is estimated that, in the last 30 years, approximately 40,000 ha of mangrove forest in Colombia has been altered by different activities, including road construction; urban growth; expansion of agriculture, fish, and livestock frontiers; and indiscriminate logging, among others [3,6]. In the case of the Colombian Caribbean region, one of the main stress factors is the alteration of their hydrology, due to the decrease in freshwater supply caused by the stream channeling, which was triggered by a progressive process of hyper salinization. This process has limited the growth of mangrove seedlings and trees for the last 10–20 years [7]. In addition, mangroves are exposed to various pollutants, including sewage and industrial effluents, along with marine and atmospheric activities [8]. In fact, many of these ecosystems are close to the mouths of rivers and receive a notable influence from contaminated water and sediments [9]. A notable example is the Magdalena River, a sink for polluted water from agricultural runoff, extensive livestock farming, mining, domestic, and industrial discharges from all over Colombia; this affects downstream ecosystems as is the case of the mangroves near its mouth. The heavy metals present are of especial relevance in this ecosystem due to their toxicity [8]. The coastal marine ecosystems of the Atlantic department are exposed to the presence of heavy metals such as Zn^2+^, Cu^2+^, Pb^2+^, Cr^6+^, Cd^2+^, and Hg^2+,^ which have been detected in sediments. This suggests a mixed origin, the consequence of anthropogenic activities (agriculture and industrial sources, together with discharge of domestic wastewater, leachates, and inputs from the Magdalena River [10]. In addition, most of these heavy metals have been detected in the tissues of fish that develop in these mangrove forests [11,12]. 

Heavy metals can be chemically defined as a group of elements with high atomic weight and density, including metals and metalloids that at low concentrations can be toxic to the environment and humans [13,14,15]. Heavy metal contamination is characterized by being covert, persistent, and irreversible, and can accumulate through the food chain, causing damage to the health of living organisms [14,16]. Metals such as Cd^2+^, Cr^3+^, Pb^2+^, and Hg^2+^, due to their high degree of toxicity, are a high priority to public health services [17]. 

In recent decades, plant-associated microorganisms have been shown to play an important role in the ability of plants to adapt to environmental disturbances [18]. In this regard, there is great interest in endophytic microorganisms for their ability to improve plant response to various biotic and abiotic stress factors [19,20]. Endophytes have successfully evolved to adapt to a heavy-metal-contaminated environment by developing responses such as biomagnification, decreased enzyme activity, promoting metal sequestering, and increased gene expression of stress responses [18,21,22]. The mechanisms used by heavy-metal-tolerant bacteria have been widely studied [23,24,25,26,27]. The bacterial strategies of tolerance and bio-transformation of heavy metals are determined genetically, and with the introduction of massive sequencing, it is possible to identify them and to verify if its presence translates phenotypically into tolerance at the laboratory level [28]. 

Many bacteria show varying degrees of multi-resistance to heavy metals when exposed to highly polluted environments, among which it is worth highlighting the genera *Pseudomonas*, *Enterobacter*, *Brevibacterium*, and *Bacillus*, among others [29]. Different species of the genus *Bacillus* such as *B. subtilis*, *B. licheniformis*, *B. cereus*, *B. clausii*, and *B. fexus* have shown high tolerance to different heavy metals [30,31,32]. The last one, *B. flexus*, recently reclassified as *Priestia flexa* [33], is a Gram-positive bacteria that is found in mangrove ecosystems [34] and shows tolerance to toxic heavy metals, among which arsenic is the most studied [35,36,37,38]. The aim of this study was to characterize the tolerance to different heavy metals, especially Hg^2+^, and detoxification capacity, of a halotolerant endophytic strain of *P. flexa* isolated from the mangrove *A. germinans* in the Colombian Caribbean. 

## 2. Materials and Methods

### 2.1. Study Area and Sampling

**Figure 1:**
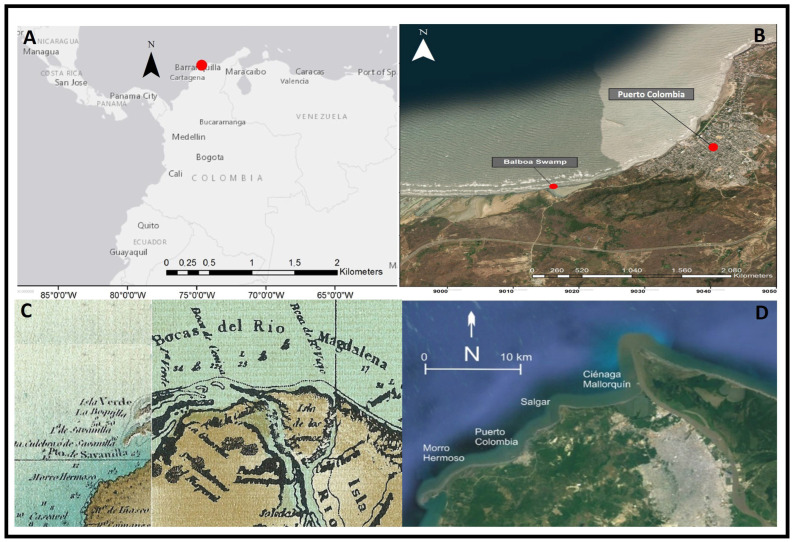
Location and evolution of the Atlántico department coastline, Caribbean coast of Colombia and Balboa Swamp. (**A**) Colombia map with sampled region marked with red; (**B**) Balboa swamp in the Colombian Caribbean coastal region; (**C**) Magdalena river delta depicted in the early 1800s; (**D**) Magdalena river delta today.

The Balboa swamp is located in the western part of the municipality of Puerto Colombia, Department Atlántico (10°58′48.12″ N, 74°58′34.50″ O) and has an area of approximately 160 hectares and a perimeter of 24,800 m, where the presence of salt marshes can be observed (Figure 1A,B) [39,40]. In the Balboa swamp, there are extensive zones in which the mangroves have disappeared, which apparently is related to the lack of fresh water [41]. The coastal strip where the Balboa swamp is located is a very dynamic area influenced by the Magdalena River, which has changed over the years (Figure 1C,D). Some physical and chemical characteristics of the water were measured with a YSI EXO-1 multiparameter probe in 6 points of the study area, and these included pH (6.57–7.16), salinity (7.04–66.97% PSU), conductivity (12.36–94.02 mS/cm), and dissolved oxygen (24.9–37.2% DO).

### 2.2. Isolation of Endophytic Bacteria from Avicennia germinans

Healthy leaves from a *Avicennia germinans* tree were sampled from Balboa swamp, (Figure 1B). Freshly acquired samples were brought to the laboratory in sterile packaging and immediately processed. Leaves were washed thoroughly with sterile distilled water, and then immersed in 80% ethanol for 1 min, followed with a wash with 4% NaOCl for 5 min. Finally, leaves were washed 8 successive times using sterile distilled water [42]. Several aliquots of the final rinse water were grown on oxytetracycline glucose agar base medium (OGYE) plates to confirm the correct surface sterilization. Healthy leaves then were macerated with 3 mL of sterile 0.9% NaCl in a sterile mortar. The macerate dilution and tissue segments were placed on OGYE agar base medium and incubated at 25 °C for 72 h [42]. Bacterial isolates were selected based on colony appearance and streaked on OGYE agar base plates until purity level was achieved. Purified isolates were grown on Luria Bertani (LB)-agar plates (Millipore^®^, Burlington, VT, USA) at 25 °C and cells were stored in 30% (*v*/*v*) glycerol at −80 °C [43]. Isolates were named X (1-7) BS3110 after the Balboa swamp.

### 2.3. Tolerance to NaCl 

Isolates were grown in tryptone soy broth (TSB) to reach the exponential phase. Next, 100 µL of each of these cultures was inoculated in TSB at different salinities (2.5%, 5%, 7.5%, 10%, 12.5%, and 15% NaCl). Cultures were incubated at 200 rpm at room temperature and growth was observed every day spectroscopically until completing 5 days of incubation. The bacteria with the greatest tolerance was selected for subsequent studies.

### 2.4. Screening of Heavy Metal Tolerance in Strain 7BS3110

Heavy metal tolerance assays were performed from a microbial inoculum of *P. flexa* incubated for 24 h in TSB. A total of 20 µL was taken and inoculated in Petri dishes with tryptic soy agar (TSA) at different concentrations (0.01, 0.1, 1, 5, 10, 20, 30, 50, 100, 250, 500, and 1000 mM) of heavy metals salts: CrCl_3_ · 6H_2_0, CuSO_4_ · 5H_2_0, ZnCl_2_, CoSO_4_ · 7H_2_O, HgCl_2_, PbNO_3_, NiCl_2_ · 7H_2_O, and CdSO_4_ in triplicate. Plates were incubated at room temperature for up to 15 days. Growth was observed at 1, 2, 3, 7, and 15 days of incubation. The last concentrations with appreciation of growth were cultivated in TSA agar without heavy metal to determine the viability of the strain. The results were expressed as minimum inhibitory concentrations (MIC) and minimum bactericidal concentrations (MBC), which correspond to the lowest level of heavy metal that inhibits growth and that causes microbial death, respectively.

### 2.5. Genomic Analyses of Strain 7BS3110

Genomic DNA was extracted using the cetyltrimethylammonium bromide-based method (CTAB) [44] and its concentration was determined with a Qubit v.2.0 fluorometer (Invitrogen, Waltham, MA, USA). Library preparation and DNA sequencing was performed by MicrobesNG (University of Birmingham, Birmingham, UK). Libraries were performed using the Nextera XT kit (Illumina, San Diego, CA, USA) following the manufacturer’s protocol. 

Sequencing was performed with Illumina MiSeq. Reads were trimmed using Trimmomatic v.0.36. [45] and quality analyses were performed with FastQC software v.0.11.8 (http://www.bioinformatics.babraham.ac.uk/projects/fastqc/, accessed on 11 January 2023). Assembly was performed with SPAdes v.3.12.0 [46]. The contigs were extended and merged into Scaffolds using SSPACE software v2.1.1 [47] and the generated gaps were closed with the GapFiller v.1-10 software [48]. The quality analyses of genome assembly were performed with Quality Assessment Tool for Genome Assemblies—Quast software v.5.2 [49] and the purity of the genome was checked using Microbial Genome Atlas v.1.3.9.0 web service (http://microbial-genomes.org, accessed on 13 February 2023) [50]. Taxonomic classification was performed using the average nucleotide identity (ANI) using both best hits (one-way ANI) and reciprocal best hits (two-way ANI) between two genomic datasets and the average amino acid identity (AAI) using both best hits (one-way AAI) and reciprocal best hits (two-way AAI) between two genomic datasets of proteins (https://enve-omics.gatech.edu/, accessed on 23 August 2023) [51,52]. Additionality, a phylogenetic tree was constructed from the 16S ribosomal DNA sequence of *P. flexa* 7BS3110 and other related reference bacteria obtained at the National Center for Biotechnology information (NCBI), which were aligned with CLUSTAL W and processed with the MEGA V 11.0.13 software using neighbor-joining method and subjected to 1.000 bootstrap replications.

The whole genome was annotated with Prokka v1.12 [53] and RAST [54]. An in-depth search for genes related to heavy metal tolerance was conducted. Therefore, a database was compiled with the sequences of the proteins related to heavy metal resistance retrieved from the UniProt database. Using this database, a search of the amino acid sequences annotated in the sequenced genome was performed using blastp. The presence of a protein was considered with a percent identity higher than 35%, a query cover higher than 50%, and an E-value smaller than 10^−5^. For proteins that met these criteria, but whose annotated name in the genome was different from that of the searched protein or corresponded to a hypothetical protein, their sequences were analyzed using the InterPro Website program [55] to establish family membership, similar molecular functions, common domains, and common cellular locations.

### 2.6. Evaluation of the Effect of Mercury on the Cell Structure of P. flexa 7BS3110

Considering the high tolerance of *P. flexa* 7BS3110 to mercury, an Erlenmeyer assay was performed with 10 mL of TSB at a concentration of 0.25 mM HgCl_2_ for 7 days. For this, an inoculum obtained from a *P. flexa* 7BS3110 colony on TSA agar was used, which was initially cultured in 10 mL of mercury-free TSB for 24 h and later 100 µL of this culture was transferred to 10 mL of TSB with 0.15 mM HgCl_2_. All the cultures were carried out at room temperature at 200 rpm and OD was measured each day at 600 nM.

### 2.7. Scanning Electron Microscope (SEM-EDX) 

A total of 200 µL from culture was taken at 2 and 4 days of incubation and fixed with 25% glutaraldehyde for one hour, then filtered on a 0.22 µm nucleopore filter with 1% saline phosphate buffer (PBS). Once filtered, 100% ethanol was added and filtered again and the filter was dried for SEM analyses.

Two types of scanning electron microscopes were used, JEOL-5600 VL, coupled to an Oxford INCA X sight EDAX energy-dispersive X-ray microanalysis and a scanning electron microscopy field-emission gun (SEMFEG) Philips XL30-FEG OL-5600. Electrically conductive carbon tabs and double sticks were pressed to conductive graphite stubs and gold-coated using a Quorum, Q150T-S. Various stubs with sample pieces were placed inside the SEM chamber in high vacuum conditions and the microstructure was analyzed. Analytical conditions were 0.2 mA current 15 kV and 10 kV accelerating voltage, for the uncoated samples and gold-coated samples, respectively.

### 2.8. Transmission Electron Microscope (TEM)

Samples were fixed in 4% paraformaldehyde and 2% glutaraldehyde in 0.1 M phosphate buffer (PB) (pH 7.2) for 2 h at room temperature and washed three times with phosphate buffer and post-fixed with 1% of OsO_4_ in water during 60 min at room temperature in the dark. Later, they were washed three times by distillate water, and incubated with 2% aqueous uranyl acetate for 1 h at room temperature, washed again, and dehydrated in increasing concentrations of ethanol between 30–90% (2 × 20 min) and 100% (2 × 30 min) at room temperature. Dehydration was finished with a mixture of ethanol/propylene oxide (1:1) for 10 min and pure propylene oxide for 3 × 10 min. Infiltration of the resin was accomplished with propylene oxide/Epon (1:1) for 45 min and pure LR white resin (London Resin Company Ltd., London, UK), overnight at room temperature. Polymerization of infiltrated samples was performed at 60 °C for 2 days. Ultrathin sections of the samples were cut using an Ultracutof Leica, and stained with uranyl acetate and lead citrate by standard procedures [56]. The samples were imaged with a JEOL JEM1400 Flash (JAPAN) and the pictures were taken to 100Kv using a One View Gatan (Pleasanton, CA, USA) 4 K × 4 K, CMSO.

### 2.9. Inductively Coupled Plasma Mass Spectrometry (ICP-MS)

To evaluate the reduction in the concentration of mercury in *P. flexa* in liquid culture, a 2 mL culture sample was centrifuged at 12,000 rpm/5 min, the supernatant was filtered with a 0.25 µm pore diameter filter to remove residual biomass, and the supernatant was used to determinate and quantify the elemental mercury content by inductively coupled plasma mass spectrometry (ICP-MS). Samples were analyzed using an ELA Samples with inductively coupled plasma mass spectrometer (ICP-MS), model NexION 2000 (PerkinElmer, Waltham, MA, USA).

### 2.10. Mercury Tolerance at Different Concentrations of NaCl

A preliminary test was carried out combining different concentrations of HgCl_2_ (0.1; 0.15; 0.2; and 0.25 mM) and NaCl (2.5%; 5%; 7.5%; and 10% *w*/*v*) in falcon tubes with 5 mL of TSB broth using an inoculum previously adapted to 0.15 mM of HgCl_2_. Tubes were incubated at room temperature at 200 rpm/9 days and from each one, 20 µL was seeded in TSA without HgCl_2_ to determine the viability of the strain. Subsequently, an Erlenmeyer test was carried out with 10 mL of TSB broth at 0.25 mM HgCl_2_ under the different concentrations of NaCl tested in the screening, for which an inoculum previously adapted to 0.15 mM HgCl_2_ was used. Cultures were incubated at 200 rpm at room temperature for 4 days and a daily sample was taken from each Erlenmeyer flask to read the optical density (600 nm) in a microplate reader.

## 3. Results

### 3.1. Screening for NaCl Tolerance of Endophytic Bacterial Isolates

The isolated bacteria were labeled 1BS3110, 2BS3110, 3BS3110, 4BS3110, 5BS3110, 6BS3110, and 7BS3110, showing a NaCl tolerance range between 7.5% and 12.5% (Table 1). Isolate 7BS3110 showed the highest tolerance to NaCl and was selected for a heavy metal tolerance and genomic analysis.

### 3.2. Screening of Heavy Metal Tolerance of Isolate 7BS3110

The tolerance to different heavy metals obtained by *P. flexa* 7BS3110 is summarized in Table 2. It is possible to observe tolerance to high concentrations of Cr^3+^ and Pb^2+^ with minimum inhibitory concentrations (MICs) of 15 mM and 10 mM, respectively, and high minimum bactericidal concentrations (MBCs) to Pb^2+^ and Co^2+^ of 1000 mM. In the case of Hg^2+^, the MIC was 0.01 mM; however, the MBC was 0.75 mM. A culture assay was performed and turbidity was observed up to 0.25 mM. *P. flexa* 7BS3110 shows low sensitivities to Ni^2+^ and Cd^2+^ with a MIC and a MBC of 5 mM and 1 mM, respectively (Table 2).

### 3.3. General Characteristics of the Genome and Taxonomic Identification of the Strain with Greater Tolerance to NaCl

To identify the genes and gene products related to heavy metal tolerance the genome of isolate 7BS3110 was sequenced. The percentages of genome completeness and contamination were 99.1% and 7.5%, respectively. General genome characteristics and assembly quality data in comparison with the genomes of other species of the genus *Priestia* are shown in Table S1 of Supplementary Material. The gene annotation resulted in a total of 4161 coding DNA sequences, 89 tRNA genes, 1 complete rRNA operon, and 11 copies of 5S rRNA. The number of genes with assigned function after the annotation was 2473, corresponding to a percentage of 57.97%. 

Based on the average nucleotide identity (ANI) and the average amino acid identity (AAI), the genome of 7BS3110 strain corresponds to the species *Priestia flexa* with a 99.3% and 95% of identity, respectively. In the Supplementary Material (Table S1 and Figure S1), the phylogenetic tree and the ANI and AAI values obtained for the different *Priestia* species analyzed are shown.

### 3.4. Identification of Heavy Metal Tolerance Proteins Sequences in P. flexa 7BS3110

According to the annotation using Prokka and Rast, *P. flexa* 7BS3110 bacterium presented a protein sequence for resistance to Cu^2+^, Zn^2+^, Cd^2+^, and Hg^2+^. In addition, protein for the synthesis and transport of siderophores, spore formation, and resistance to stress conditions were also detected. In Table S2 of the Supplementary Material, the proteins found in the bibliographic review associated with the resistance of Hg^2+^, Pb^2+^, and Cr^3+^ are detailed; these are summarized in Table 3. In the case of Hg^2+^, proteins for the synthesis of EPS and bacillithiol were identified and in relation to the protein codified by *mer* operon, only the sequences of MerA and MerR were identified. The sequence of SmtA and SmtB proteins, related to Hg^2+^ bioaccumulation, are not evident; however, a protein sequence with similar family, domain, and function to ArsR/SmtB regulator was found. In the case of Pb^2+^, there are no specific protein binding sequences to this metal for its efflux, and only a similar function protein to pbrA was found. Other non-specific transporters and efflux regulators reported to Pb^2+^ efflux such as CadA and CadC were identified, and many protein sequences associated with siderophores transport were found. For Cr^6+^, a similar sequence of the proteins ChrA and ChrB, which are associated with its efflux, were identified; however ChrR, a protein related to its reduction, could not be found. However other oxidoreductases like NfsA, NfsB, AzoR, and NemA were identified. Likewise, some proteins associated with Cr^6+^ resistance, due to processes related to sulfur or iron homeostasis, like CysC, CysL, and CysP, were found (Table 3).

### 3.5. Effect of Hg^2+^ on Cell Morphology and Growth of P. flexa 7BS3110

**Figure 2:**
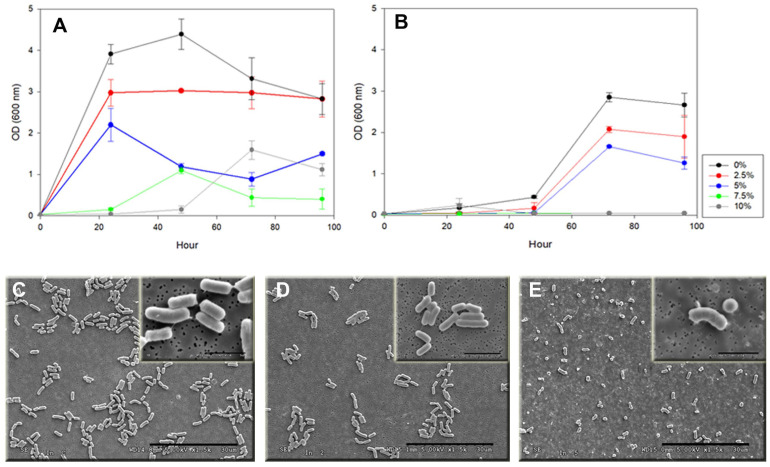
Effect of Hg^2+^ on cell morphology and growth of *P. flexa* 7BS3110 (**A**) Controls at different concentrations of NaCl without Hg^2+^. (**B**) Growth in the presence of 0.25 mM Hg^2+^ at different concentrations of NaCl. (**C**) SEM image of cells in culture without Hg^2+^. (**D**) SEM image of cells with Hg^2+^ (0.25 mM) at 2 days. (**E**) SEM image of cells with Hg^2+^ at 7 days.

The effect of Hg^+2^ on cell morphology, evidenced by SEM analysis, and on microbial growth, through the measurement of OD over time, is shown in Figure 2. In relation to the growth of this bacterium at different concentrations of NaCl and HgCl_2_, growth was only observed with 2.5% of NaCl at all HgCl_2_ concentrations tested. However, when viability was determined, colonies were obtained in all the combinations tested. Then, we proceeded to evaluate the growth at the maximum concentration of HgCl_2_ tested with different concentrations of NaCl. The results show that *P. flexa* 7BS3110 was only able to grow at 2.5 and 5% of NaCl with 0.25 mM of HgCl_2_ from the second day (Figure 2B), unlike the controls without mercury, where growth was observed at all concentrations evaluated from the first day of growth at these same concentrations; at 7.5% and 10%, the bacteria began to grow on the second and third day, respectively (Figure 2A).

At cell level, the effects of exposure to mercury in *P. flexa* 7BS3110 were observed by SEM (Figure 2C–E), from which it was possible to detect changes in the morphology of the cells after 7 days of exposure, observing abundant coccus-shaped cells, which were very different from the bacillary morphology of the untreated controls. This morphology was still observable after two days of incubation (Figure 2E). 

### 3.6. Capacity to Reduce the Concentration of Hg^2+^ in Solution by P. flexa 7BS3110

**Figure 3:**
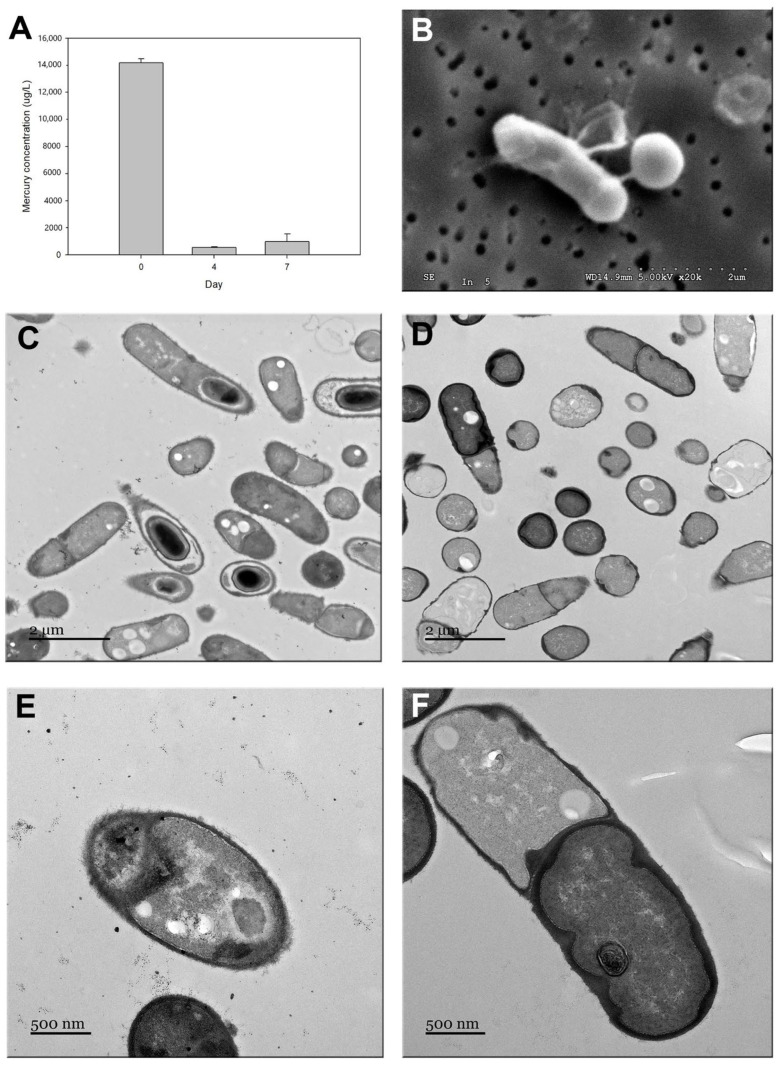
Reduction and extracellular sequestration of Hg^2+^ by *P. flexa 7BS3110*. (**A**) Hg^2+^ concentration in solution at different incubation times. (**B**) SEM image showing the presence of EPS. (**C**) TEM image of cells grown without Hg^2+^. (**D**) TEM images of cells grown in the presence of 0.25 mM Hg^2+^. (**E**) TEM image of cells grown in the absence of Hg^2+^. (**F**) TEM images of cells grown in the presence of 0.25 mM Hg^2+^.

Figure 3 shows the ability to sequester mercury in *P. flexa* 7B3110 by measuring Hg^2+^ in the culture medium and through SEM and TEM observations. The incubation of *P. flexa* 7BS3110 in the presence of 14 mg/L of Hg^2+^ resulted in a reduction of 96% of Hg^2+^ from the solution (*p* = 0.05 in U Mann–Whitney test) after four days (Figure 3A). A slight increase in the concentration of Hg^2+^ was observed after seven days of incubation (Figure 3A). Additionally, the TEM observations of P. flexa 7B3110 cells exposed to Hg^2+^ showed an electrodense layer around the cell (Figure 3D,F), which was absent in the unexposed bacteria (Figure 3C,E). The SEM images show the exudation of exopolysaccharides (EPS) that could lead to biofilm formation (Figure 3B). 

## 4. Discussion

The Balboa swamp (Figure 1B,C) arose as the result of anthropogenic activity at the mouth of the Magdalena River [7,85,86]. In 1935, the breakwaters known as “Tajamares de Bocas de Ceniza” were built at the mouth of the Magdalena River, which favored severe erosion on the west side of the delta (Figure 1D). In the following decades, these sediments, together with their vegetation, merged with the coastline and formed different swamps, among them the Balboa swamp, which, at present, is undergoing a process of hypersalinization due to the decrease in freshwater supply [7,85,86,87]. Recently, different studies have detected the presence of Cd^2+^ (0.46 µg/mL), Cr^6+^ (<5 µg/mL), Ni^2+^ (2.68 µg/mL), Cu^2+^ (2.47 µg/mL), and Pb^2+^ (0.12 µg/mL), in both water and sediments [88,89], and in the case of Hg^2+^ (0.18 µg/g), in the sediments [10]. Therefore, the mangrove forests found on the coast of the department of Atlántico (e.g., Balboa swamp) have been in contact, throughout their evolutionary history, with all types of pollutants transported by the waters and sediments of the Magdalena River. Currently, these mangroves are under increasing anthropogenic pressure, mainly due to industrialization and urbanization [3,90]. In mangrove ecosystems, heavy metals are deposited in the sediments, where they bind to iron oxides and are transferred to the mangrove plant [91]. In this regard, mangrove plants are recognized as heavy metal bioaccumulators [92,93,94]. In mangrove plants, these metals have been detected in roots and in leaves [94,95]. Therefore, in response to heavy metal and saline pressure, many microorganisms associated with mangrove plants have developed mechanisms to counteract these stressors. 

Of the isolates obtained in this study, *P. flexa* 7BS3110 was the endophytic bacteria that had the highest NaCl tolerance, exhibiting the greatest capacity to grow in the presence of sodium chloride (12.5% *w*/*v*) and, as such, can be categorized as a halotolerant bacterium [96]. As shown in Table 4, other researchers have demonstrated that different mangrove species located in various parts of the world serve as sources of halotolerant microorganisms [97]. In fact, it is worth highlighting, that in our case, this is the first recorded strain of *Prexia flexa* identified as a halotolerant endophyte in *A. germinans* from the Colombian Caribbean. In addition, it is more tolerant to NaCl than any other specimens examined in an estuarine ecosystem [98,99,100,101,102,103]. In relation to heavy metal exposure, the obtained results with the polyresistant *P. flexa* 7BS3110 show its tolerance to higher concentrations than other described microorganisms, such as *Cupriavidus metallidurans* CH34^+^, a polyresistant bacterium used in many tolerance studies as a positive control [104], or other endophytic bacteria, especially with regard to Pb^2+^, Hg^2+^, Cr^+3^, and Ni^2+^ [105,106,107]. According to the data in Table 2, the MICs of *P. flexa* 7BS3110 were higher for some heavy metals, despite having been isolated from a mangrove ecosystem apparently less exposed to heavy metal contamination. 

In the case of mercury, there are few studies of tolerance in endophytic bacteria to this extremely toxic metalloid (Table 4); in one of them, this was lower than those shown by the *P. flexa* 7BS3110 strain evaluated in this study [108,109]. The effects of exposure to high concentrations of mercury on *P. flexa* 7BS3110 became evident with the morphology changes observed with SEM (Figure 2E). This phenomenon of cell size change has also been reported in a strain of *Baccillus pumilus* exposed to different heavy metals [110]. On the other hand, when *P. flexa* 7B3110 was grown in the presence of Hg^2+^ and NaCl, its ability to tolerate Hg^2+^ up to 5% (*w*/*v*) of NaCl was observed. This can be considered a consequence of its exposure within the mangrove ecosystem to both stressors in the Balboa swamp. Few studies have evaluated the exposure of both extruders, however, the described tolerance ranges to Hg^2+^ in the presence of NaCl are in the range of 5–10% (*w*/*v*) [111,112]. In fact, halotolerant bacteria resistant to mercury and lead from hypersaline lakes in Nigeria have been reported [113].

Numerous are the mechanisms that bacteria employ to tolerate high heavy metal concentrations. In fact, some are common, among them efflux, intracellular bioaccumulation, extracellular sequestration, and biotransformation [28]. In the case of mercury, the main mechanism is its enzymatic reduction once it has entered the cell through a series of transporters, all of which are encoded by the *mer* operon [67]. However, according to the analysis of the *P. flexa* 7BS3110 genome, it was found to possess only the enzymes MerA and the MerR regulator, but no transporters for inorganic mercury such as MerT could be identified (Table 3). The simplicity of the *mer* operon found in *P. flexa* 7BS3110 is underscored by the fact that primitive bacteria of the order *Aquificales* only harbor the *merA* gene at the chromosomal level, and its complexity has evolved through horizontal gene transfer to enhance the Hg^2+^ detoxification system within the Mer system [114]. In fact, genes like *merG* and *merB*, responsible for methylmercury degradation, constitute a system that is less common in bacteria possessing *merA* [114]. In this context, the full capability for mercury volatilization within this endophytic bacterium is limited, leading to the assumption that the heightened tolerance of this microorganism to this metalloid may be attributed to extracellular sequestration, as suggested by the performed TEM analysis of *P. flexa* 7BS3110 (Figure 3D,F).

Among the extracellular sequestration strategies for mercury, binding to bacterial exopolysaccharides (EPS) has been reported [115]. The EPS possess functional groups with charges that confer adsorptive and adhesive properties [116]. The ability to synthesize EPS by *P. flexa* (previously *Bacillus flexus*) has been reported in a strain isolated from marine sediments in the Mediterranean. Characterization of this strain revealed that its EPS are composed of at least 24% sulfate, predominantly featuring glucuronic acid, galacturonic acid, glucose, and mannose [117]. In our study, many of the protein sequences for EPS synthesis reported for the *Bacillus* genus [66] were found in *P. flexa 7*BS3110 (Table 3 and Table S2 of Supplementary Material). These proteins include EpsA and EpsB as regulators and determining polysaccharide chain length, EpsD for chain elongation, EpsG and EpsF for repeat synthesis of sugar units, and the EpsM for polymerization and/or secretion. Although *P. flexa* 7BS3110 does not have all the proteins reported in the synthesis of exopolysaccharides, it has some of the key proteins associated with each phase of the EPS production process [66]. In this sense, mercury can interact with carbohydrate-rich EPS and their carboxyl, phosphoryl, and sulfhydryl groups [118,119], This could be a possible mechanism by which mercury binds to the cell structure of *P. flexa* 7BS3110, thus removing it from solution. On the other hand, the absence of metallothionein sequences, such as the SmtA protein responsible for intracellular sequestration in *P. flexa* 7BS3110 (Table 3 and Table S2 of Supplementary Material) and the absence of electrodense zones within the cell through TEM analysis (Figure 3D,F), rule out a possible intracellular accumulation mechanism. In fact, according to the literature, the percentage of mercury-resistant bacteria isolated from marine environments utilizing this mechanism is rather low [120]. 

In the case of lead, the presence of protein sequences leading to EPS synthesis can also be associated with *P flexa* 7BS3110’s tolerance to this metal, since a specific binding of this metal to EPS has been reported [121,122,123]. Therefore, protein sequences linked to the synthesis of siderophores that are correlated with the resistance to this metal and its transport were sought in the literature [79,124]. Due to the identification of only the sequences of enzymes associated with the recognition and transport of siderophores, this mechanism can be ruled out (Table 3 and Table S2 of Supplementary Material). Additionally, no lead-specific binding proteins were identified for the efflux mechanisms (Table 3 and Table S2 of Supplementary Material), despite the fact that these mechanisms are known for their high specificity to this metal [77,125]. The presence, however, of the P-type ATPase transporter sequence CadA and its regulator CadC, could suggest that the mechanism employed by *P. flexa* 7BS3110 to tolerate high Pb^2+^ concentrations is probably efflux, facilitated by these transporters (Table 3 and Table S2 of Supplementary Material). Although recognized primarily for cadmium transport, these transporters have also been shown to be involved in the cellular efflux of lead [126,127,128]. 

As for *P. flexa* 7B3110 Cr^3+^ resistance, although many studies have focused on bacteria Cr^6+^ resistance, recently, the Cr^3+^ toxicity on *Bacillus subtilis* and *Escherichia coli* has been evaluated, showing effect on growth and membrane cell at 0.19 mM of Cr^3+^ [84]; in addition, some environmental isolates have shown tolerance around 28 mM of trivalent chromium [129]. However, considering that Cr^3+^ and Cr^6+^ can be interconverted by oxidation or reduction reactions [130], in this study, we considered the mechanisms for both Cr^3+^ and Cr^6+^ tolerance. The presence of a specific efflux mechanism like the ChrA protein, an efflux protein driven by the membrane potential, and ChrB, a chromate-sensing regulator, could facilitate its extrusion [83,131]. However, it has been reported that the presence of this mechanism only cannot explain by itself the Cr^6+^ resistance [83]. In this sense, in the case of *P. flexa* 7BS3110, its high Cr^3+^ resistance could be also due to the presence of different reductases sequences, such as NfsA, NfsB, AzoR, and NemA, because different studies have shown that these reductases have chromate reductase as a secondary function [132,133]. Likewise, we only identified three protein sequences, CysC, CysL, and CysP, associated with sulfur homeostasis, which have also been related to chromium resistance [134]. The Sod protein sequence, a superoxide dismutase able to counteract oxidative stress upon exposure to ROS [130], was identified; it is a mechanism present in chromium-resistant bacteria to avoid heavy-metal toxicity [135]. 

The moderate resistance to the other heavy metals of the 7BS3110 isolate (Table 1 and Table S2 of Supplementary Material) is probably related to the presence of protein sequences for the synthesis of bacillithiol compounds. Thiols are compounds that play a role in detoxifying metal stressors; in fact, they have been linked to the detoxification of mercury through binding to this metal prior to its reduction [114]. However, in the case of the endophytic strain *P. flexa* 7BS3110, it is possible that the mercury does not enter the cell, so the role of thiol must be focused on buffering and metal ion homeostasis, thereby safeguarding cells from metal ion intoxication [74,136].

Finally, *P. flexa* 7BS3110 was able to remove 96% of a medium with of 14 mg/L of Hg^2+^ after 4 days of incubation (Figure 3A). The variations in the removal efficiency depend on the strain used and the tested medium [137]. In this respect, a strain of *Bacillus* spp., isolated from heavy-metal-contaminated soils, demonstrated a Hg^2+^ reduction effectiveness ranging from 62% to 97% in a medium with 10 mg/L of the metalloid [138]. In addition, bacteria isolated from wastewater, such as *Stenotrophomonains maltophilia* and *Pseudomonas stutzeri*, achieved removal efficiencies close to 100% at a solution of 20 mg/L de HgCl_2_ [139]. In turn, the percentage of Hg^2+^ bioaccumulation of *Acinetobacter junii* and *Pseudomonas stutzeri* was 76% and 90%, respectively, when incubated in the presence of 50 mg/L of Hg^2+^ [140]. According to these results, it can be concluded that the reduction percentage obtained in this study falls within a competitive range with previous studies, suggesting that the endophytic strain *P. flexa* 7BS3110 exhibits high mercury removal efficiency. Furthermore, *P. flexa* 7BS3110 can remove Hg^2+^ from solution in the presence of 5% (*w*/*v*) NaCl. This ability has also been reported for *Pseudomonas aeruginosa*, isolated from Keputih non-active sanitary landfill leachate, Surabaya, Indonesia, which was able to eliminate mercury with a percentage close to 100% at a 10% salinity when incubated in the presence of 10 mg/L of Hg^2+^ [141]. 

## 5. Conclusions

Mangrove ecosystems are recognized for their high resilience to natural phenomena and anthropogenic activities, which makes them a strategic ecosystem for climate change adaptation. Endophytic microorganisms are characterized by promoting the growth of their host plant under biotic (e.g., resistance to phytopathogens) and abiotic (e.g., salinity, drought, and heavy metals) stress conditions. 

In our pioneering study in the Colombian Caribbean, we identified a halotolerant endophytic strain of the mangrove *Avicennia germinans* identified as *Priestia flexa* 7BS3110. This strain not only proved to be able to resist up to 12.5% NaCl, but also stands out for its multi-resistance to heavy metals such as Pb, Cr, and Hg. In addition, its ability to sequester mercury from the culture medium has been demonstrated.

The potential of *P. flexa* 7BS3110 for bioremediation is particularly remarkable, due to its ability to resist heavy metals and high salt concentrations. This makes it an excellent candidate for programs aimed at recovering and restoring aquatic ecosystems including freshwater bodies, seawater, and estuaries contaminated by heavy metals. In addition, *P. flexa* 7BS3110 could be used in the bioremediation of hypersaline soils in terrestrial ecosystems promoting sustainable agricultural practices that improve crop productivity in soils affected by salinity and/or heavy metals.

Considering that mangroves are resilient ecosystems and sentinels of climate change, it is necessary to continue researching and searching for alternatives for bioremediation, bioprospecting, biofertilization, biocontrol, and the improvement of agricultural activities. These ecosystems host microorganisms with unique capabilities that can favor environmental restoration by mitigating the adverse effects of anthropogenic activities and climate change. Although this study reports a strain with potential agrobiotechnological applications, further studies on microbial diversity and its interactions with mangrove plants are needed, to provide a better understanding of the mechanisms underlying mangrove resilience and the potential for innovative biotechnological applications.

In summary, the findings on the endophytic strain *P. flexa* 7BS3110 highlight the relevance of mangrove endophytic microorganisms and the valuable contributions they can offer to environmental and agricultural biotechnology by providing alternatives with which to address global environmental challenges.

## Figures and Tables

**Table 1:** Characteristics of the endophytic bacteria isolated from *A. germinans*.

Isolates	Gram Stain Result and Morphology	Maximum Concentration NaCl Tolerance (%)
**1BS3110**	Gram-stain-positive bacilli	7.5
**2BS3110**	Gram-stain-positive bacilli	10
**3BS3110**	Gram-stain-positive cocci	7.5
**4BS3110**	Gram-stain-positive cocci	10
**5BS3110**	Gram-stain-negative bacilli	10
**6BS3110**	Gram-stain-positive cocci	10
**7BS3110**	Gram-stain-positive bacilli	12.5

**Table 2:** LD50 of different heavy metals and MIC and MBC of *Priestia flexa* 7BS3110.

Heavy Metals						
Name	Compound	LD50 (mg/Kg)	Ref.	*P. flexa*		Ref.
				MIC (mM)	BMC (mM)	
Chrome	CrCl_3_ · 6H_2_0	1790	[57]	15	50	This study
Copper	CuSO_4_ · 5H_2_0	384	[58]	5	30	
Zinc	ZnCl_2_	528	[59]	1	30	
Cobalt	CoSO_4_ · 7H_2_O	450	[60]	5	1000	
Mercury	HgCl_2_	0.25–2.25 kg	[61]	0.01	0.75	
Lead	Pb(NO_3_)_2_	2250	[62]	10	1000	
Nickel	NiCl_2_ · 7H_2_O	500	[63]	5	5	
Cadmium	CdSO_4_	200	[64]	1	1	

**Table 3:** Proteins associated with Hg^2+^, Pb^2+^, Cr^3+^, and Cr^6+^ resistance in *P. flexa* 7BS3110.

Mercury					
Activity	Protein Product	Gene	Protein Code	Similarity Protein Results	Ref.
Extracellular sequestration	Exopolysaccharide biosynthesis EpsA	*epsA*	AEP92472.1	SFu, SDo, SCu	[65,66,67]
	Tyrosine kinase	*epsB*	WFA11311.1	Presence	[66,67,68]
	Glycosyltransferase EpsD	*epsD*	SIQ94561.1	Presence	[66,67,68]
	Glycosytransferase EpsF	*epsF*	CAH0138532.1	Presence	[65,66,67]
	Exopolysaccharide biosynthesis EpsG	*epsG*	WFA11306.1	Presence	[65,66,67]
	Putative sugar transferase EpsL	*epsL*	CAH0206043.1	SFu, SDo, SCu	[65,66,67]
	Acetyltransferase EpsM	*epsM*	SIQ94782.1	Presence	[66,67,69]
	Aminotransferase EpsN	*epsN*	SIQ94819.1	Presence	[66,67,69]
	Transcriptional regulator SinR	*SinR*	KFM97474.1	Presence	[66,67,70]
	Wzz N-terminal domain	*Wzz*	WP_049163267.1	SFu, SDo, SCu	[65,67]
*mer* Operon	Mercuric reductase	*merA*	WP_138116160.1	Presence	[67]
	Broad mercury transporter merE	*merE*	BAS29549.1	SCu	[67]
	Mercuric transport	*merP*	QCS51148.1	SFa, SDo, SFu	[67]
	Mercuric resistance operon	*merR*	WP_289521299.1	Presence	[67]
Intra cellular accumulation	Metalloregulator smtB	smtB	WP_195783159.1	SFa, SDo, SFu	[71]
Thiol	L-malate glycosyltransferase BshA	*bshA*	WP_210608563.1	Presence	[28,72,73,74]
	Glucosaminide deacetylase BshB1	*bshB1*	AIC44925.1	Presence	[28,72,73]
	Glucosaminide deacetylase BshB2	*bshB2*	WEZ07453.1	Presence	[28,72,73,74]
	Bacillithiol synthase	*bshC*	WP_034649136.1	Presence	[28,72,73,74]
**Lead**					
**Activity**	**Protein Product**	**Gene**	**Protein code**	**Similarity protein results**	**Ref.**
Specific Efflux	Pb-efflux protein PbrA	*pbrA*	CAI11271.1	SFa, SDo, SFu, SCu	[75,76]
Non-specific efflux	Transcriptional regulator CadC	*cadC*	CAH0174756.1	Presence	[29]
	Transcriptional regulator CmtR	*cmtR*	WP_308474560.1	SFa, SDo, SFu	[77]
	ATP-binding protein AztA	*aztA*	WP_078614428.1	SFa, SDo, SFu	[77]
	Metalloregulator AztR	*aztR*	WP_042466054.1	SFa, SDo, SFu	[77]
	Cation efflux P1-ATPase	*czcP*	ABF12829.1	SFa, SDo, SFu, SCu	[77]
	Transporting Cd^2+^, Zn^2+^, Co ATPase	*cadA*	OFC98076.1	Presence	[29]
Intra cellular accumulation	Metalloregulator SmtB	*smtB*	WP_195783159.1	SFa, SDo, SFu	[71,76]
Siderophores	Lysine decarboxylase DesA	*desA*	WP_302000531.1	SFa	[76,78,79]
	Cadaverine hydroxylase	*desB*	AOC37727.1	SFa	[76,78,79]
	Alcaligin biosynthesis enzyme	*alcA*	AAA97596.1	SFa	[76,79,80]
	Acetyltransferase	*alcB*	WP_013084704.1	SFa, SDo, SFu	[76,79,80]
	Siderophore transport	*STra*	QLK07663.1	SFa, SDo, SFu, SCu	[76]
	Siderophore related permease	*Sperm*	QLK04956.1	SFa	[76]
	Siderophore transport system	*FeSB*	WP_055992581.1	SDo, SUc	[76]
	Siderophore transport system	*FeP*	CBS89604.1	SFa, SFu, SCu	[76]
	Siderophore transport system	*FeATP*	SPR94826.1	SFa, SDo, SFu	[76]
	Siderophore transport system	*ABCp*	QLK08254.1	SFa, SFu, SCu	[76]
	Siderophore transport system	*ABCatp*	QLK04041.1	SFa, SDo, DFu	[76]
	Siderophore transport system	*ABCsb*	QLK04038.1	SDo, SUc	[76]
	Siderophore transport system	*ABCp2*	QLK04040.1	SFa, SFu, SCu	[76]
**Chrome**					
**Activity**	**Protein Product**	**Gene**	**Protein code**	**Similarity protein results**	**Ref.**
Efflux	Chromate resistance efflux	*chrA*	GMG74543.1	SFa, SFu, SCu	[81,82]
	Chromate efflux transporter	*chrB*	GMG74544.1	SFa, SFu, SCu	[81,82]
Sulfur or iron homeostasis	Adenylyl sulfate kinase	*cysC*	WKU24108.1	Presence	[82,83]
	Sulfite reductase	*cysL*	KNH16506.1	Presence	[82,83]
	Ferritin	*fntA*	WP_283880548.1	SDo	[82,83]
	Sulfate/thiosulfate import	*CysA*	CAH0220131.1	SFa, SDo	[82,83]
	Sulfate transporter permease	*CysT*	WP_013082952.1	Presence	[82,83]
Reduction of chromium VI to chromium III	NADPH nitroreductase	*NfsA*	WP_028412760.1	SFa, SDo, SFu	[83]
	NAD(P)H oxidoreductase	*NfsB*	WP_013083136.1	SFa, SDo, SFu, SCu	[83]
	N-ethylmaleimide reductase	*NemA*	CAH0175675.1	Presence	[83]
	NADH-azoreductase	*AzoR*	ADF40836.1	Presence	[83]
	NADPH nitroreductase	*Frp*	WP_098524731.1	SFa, SDo, SFu	[83]
	Oxidoreductase	*YcnD*	AEN89027.1	SFa, SDo, SFu	[83]
	AD(P)H oxidoreductase	*NfoR*	WP_000069098.1	SFa, SDo, SFu, SCu	[83]
Oxidative stress reduction	Thioredoxin	*trxA*	WP_277717273.1	Presence	[83]
	Glutaredoxin	*grxA*	PVE91831.1	SFa	[83]
	Superoxide dismutase SodA	*sodA*	WP_195674987.1	Presence	[84]

SFa: similar family protein, SFu: similar function, SDo: similar domain; SCu: similar cellular ubication.

**Table 4:** Tolerance to NaCl and heavy metal of endophytic bacteria shown in others works.

Bacteria	Compound/ Metal	Tolerance	Source	Ref.
*Bacillus halotolerans*	NaCl	3.2% (*w*/*v*)	*Avicennia germinans*	[98]
*Ancylobacter mangrovi* sp.	NaCl	7% (*w*/*v*)	*Bruguiera gymnorrhiza* and *Sonneratia apetala*	[99]
*Bacillus altitudinis*	NaCl	8% (*w*/*v*)	*Acanthus ilicifolius* L.	[100]
*Acinetobacter* (Pseudomonadota)	NaCl	5% (*w*/*v*)	*Avicennia marina* propagules	[101]
*Staphylococcus* (Bacillota)	NaCl	10% (*w*/*v*)	*Avicennia marina* propagules	[101]
*Staphylococcus* (Bacillota)	NaCl	10% (*w*/*v*)	*Avicennia marina* propagules	[101]
*Salinicola salaries*	NaCl	10% (*w*/*v*)	*Avicennia officinalis* L.	[102]
*Brachybacterium halotolerans* sp.	NaCl	20% (*w*/*v*)	*Bruguiera gymnoirhiza*	[103]
*Bacillus* sp.	Pb2^+^	MIC of 4 mM	*Solanum nigrum* L.	[105]
	Cr^6+^	MIC of 12 mM		
*Serratia* sp.	Pb^2+^	MIC of 4 mM	*Solanum nigrum*	[106]
	Cr^6+^	MIC of 12 mM		
*Bacillus* sp.	Pb^2+^	MIC of 3.6 mM	*Commelina communis*	[107]
	Cu^2+^	MIC of 1.6 mM		
	Cd^2+^	MIC of 0.9 mM		
Not specified	Hg^2+^	MIC of up to 5 mM	*Aeschynomene fluminensis* and *Polygonum acuminatum*	[108]
*Bradyrhizobium* spp.	Hg^2+^	MIC > 150 μM	*Calicotome spinosa* roots	[109]

## Data Availability

Data are contained within the article and Supplementary Materials.

## Supplementary Materials

The following supporting information can be downloaded at: https://www.mdpi.com/article/10.3390/microorganisms12091857/s1, Table S1. Genome characteristics and assembly quality of *P. flexa* 7B3110 and other species of the genus *Priestia*. Figure S1. Phylogenetic tree of 16S ribosomal RNA sequences of *P. flexa* 7BS310 and other related species. Table S2. Analysis of the presence of protein sequences associated with Hg^2+^, Pb^2+^, Cr^3+,^ and Cr^6+^ resistance in the genome of *P. flexa* 7BS3110.
