# Supplementary material for: Halotolerant Endophytic Bacteria Priestia flexa 7BS3110 with Hg2+ Tolerance Isolated from Avicennia germinans in a Caribbean Mangrove from Colombia
Source: Microorganisms. 2024 Sep 7;12(9):1857. doi: 10.3390/microorganisms12091857 (PMC11434322; doi:10.3390/microorganisms12091857)

microorganisms
MDPI

Figure S1: phylogenetic tree of 16S ribosomal RNA sequences of *P. flexa* 7BS310 y other and other associated species.

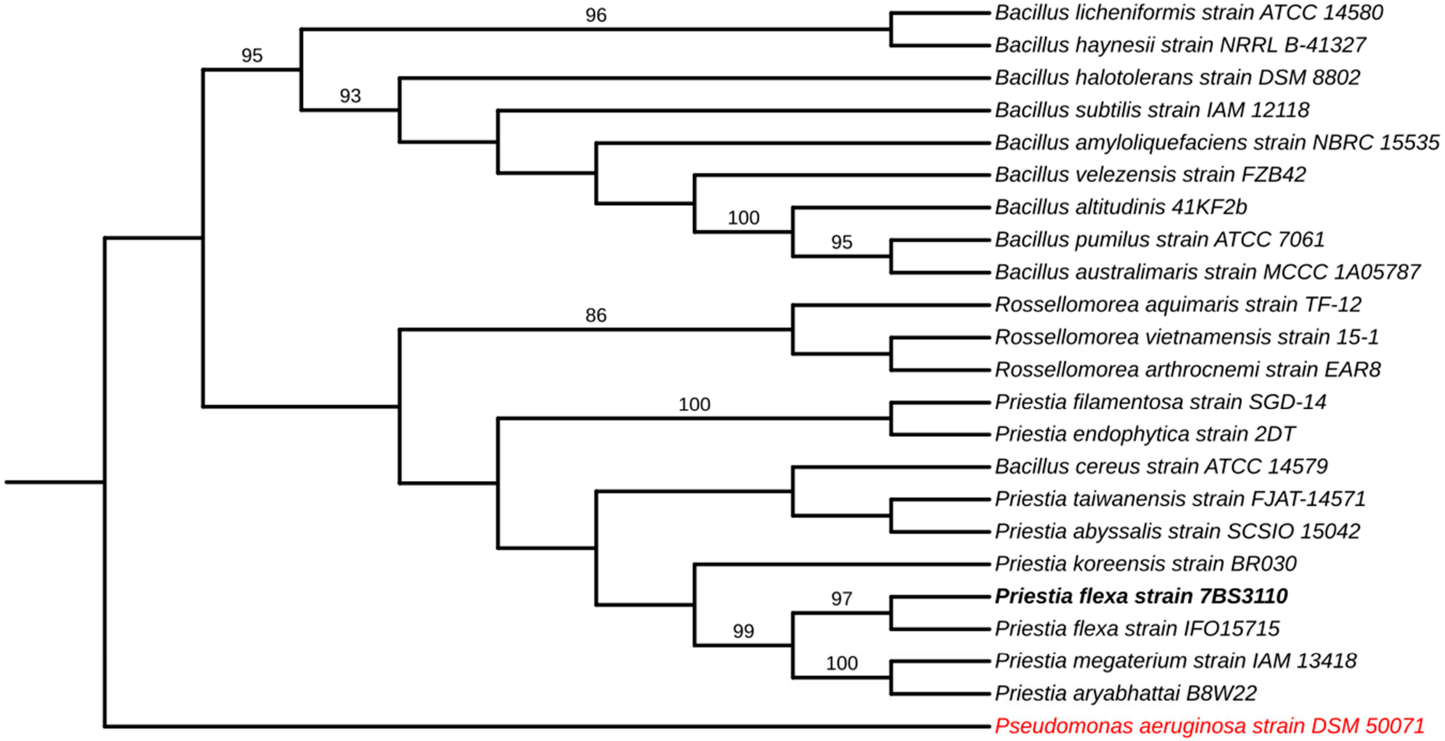

Supplement: Supplementary file 1 [file microorganisms-12-01857-s001.zip › Supplementary Material Figure S1 240524.pdf]
